# Adverse childhood experiences and dental anxiety among Chinese adults in Hong Kong: a cross-sectional study

**DOI:** 10.3389/fpsyg.2024.1372177

**Published:** 2024-05-22

**Authors:** Natalie Sui Miu Wong, Andy Wai Kan Yeung, Colman Patrick McGrath, Yiu Yan Leung

**Affiliations:** ^1^Oral and Maxillofacial Surgery, Faculty of Dentistry, The University of Hong Kong, Pokfulam, Hong Kong SAR, China; ^2^Applied Oral Sciences & Community Dental Care, Faculty of Dentistry, The University of Hong Kong, Pokfulam, Hong Kong SAR, China

**Keywords:** dental anxiety, adverse childhood experiences, childhood adversity, cross-sectional study, modified dental anxiety scale

## Abstract

**Introduction:**

This study aimed to investigate the relationship between adverse childhood experiences (ACEs) and dental anxiety among Chinese adults in Hong Kong.

**Methods:**

A cross-sectional survey was conducted at a university in Hong Kong. The recruiting period and data collection started in January 2023 and ended in June 2023. Participants completed an online questionnaire that assessed ACEs (using the Adverse Childhood Experiences International Questionnaire – ACE-IQ) and dental anxiety (using the Modified Dental Anxiety Scale – MDAS and Dental Fear Survey – DFS). The study examined the impacts of both cumulative (i.e., total number) and independent ACE components on dental anxiety. To explore the relationships between cumulative ACEs, individual ACE components and dental anxiety (MDAS and DFS score), Pearson correlations, linear regression and logistic regression were conducted.

**Results:**

Significant associations were observed between ACEs and dental anxiety among 171 subjects. Cumulative ACEs were positively correlated with MDAS scores (*r* = 0.169, *p* = 0.027) and DFS scores (*r* = 0.253, *p* < 0.001). The odds of an individual having high dental anxiety increased by 26–43% for every additional increase in the number of ACEs. Individual types of ACEs, such as emotional and physical neglect, sexual abuse, and household substance abuse, significantly influenced the likelihood of having high dental anxiety.

**Discussion:**

The results showed a positive association between ACEs and dental anxiety, highlighting the impact of ACEs on dental anxiety. Dental practitioners should consider inquiring about a patient’s ACE history to develop personalized treatment plans.

## 1 Introduction

Dental anxiety has been a topic of investigation for over two centuries, with its historical roots traced back to the late 1800s and early 1900s (Yeung, 2023). The terms ‘dental fear,’ ‘dental anxiety,’ and ‘dental phobia’ are often used interchangeably, but they refer to distinct mental states with different clinical features. Fear is characterized as a response to immediately threats, triggering a high state of arousal and activating the sympathetic nervous system, which prepares individuals for a “flight-or-fight” response. Meanwhile, anxiety involves apprehension about anticipated future threats, with lower arousal compared to fear. Individual experiencing anxiety may feel tense and restless. Specific phobia, categorized as an anxiety disorder in the Diagnostic and Statistical Manual of Mental Disorders, fifth edition (DSM-5) (American Psychiatric Association, 2013) and the International Classification of Disease, eleventh revision (ICD-11) (World Health Organization, 2019), is characterized by excessive and disruptive fear of a particular object or situation. The exaggerated fear experienced in dental phobia is irrational and disproportionate to the actual danger posed. Individuals with phobias may acknowledge the excessive and unrealistic nature of their fear but struggle to control their preoccupation with the feared object or situation. Avoidance of distressing situations is common among people with phobias and can significantly impair their daily functioning (Kring et al., 2007; American Psychiatric Association, 2013; Kring and Johnson, 2018; World Health Organization, 2019).

Dental anxiety and dental phobia are not classified in DSM-5 or ICD-11. Some researchers have suggested that dental phobia can be considered as an example of specific phobia, but it is unclear whether it should be grouped under the blood–injection–injury (BII) subtype (300.29) in DSM-5 or as an independent subtype (Kılıç et al., 2014; Van Houtem et al., 2014). The prevalence of dental anxiety has been investigated in various populations over the past few decades and has been shown to be common and persistent in general population. A recent meta-analysis found that the prevalence of dental anxiety among preschoolers, schoolchildren and adolescents was 36.5, 25.8, and 13.3%, respectively, (Grisolia et al., 2021). Another recent meta-analysis reported a global prevalence of dental anxiety among adults to be 15.3% (Silveira et al., 2021), indicating that dental anxiety is high across different age groups worldwide.

Dental fear is a complex and multifactorial phenomenon that can set the stage for the development of dental anxiety or dental phobia. Several risk factors have been found to increase the likelihood of developing dental anxiety or dental phobia, some of which are modifiable or controllable while others are not. Modifiable factors include the influence of family members, friends and media (Wong et al., 2022), negative attitude from dental practitioners, perceptions of dentists’ behavior, communication style and competence (Corah et al., 1985; Abrahamsson et al., 2002), feelings of a lack of control and unpredictability (Rouse and Hamilton, 1990), fear of dental surgery (Oosterink et al., 2008), and anxiety-provoking stimuli such as the sound of dental drills, the act of injections, the sight of needles, and the smells of the clinic (Moore et al., 1993; Oosterink et al., 2008; Wong et al., 2022). Non-modifiable factors include a history of psychological disorders (Locker et al., 2001), previous traumatic dental experiences (Murad et al., 2020), personality traits such as introversion and neuroticism (Schuurs et al., 1988; Halonen et al., 2012), a lower pain threshold, and a higher sensitivity to pain (Klages et al., 2004). Other non-modifiable factors such as genetics, gender, and age also contribute to dental anxiety (Ray et al., 2010; Appukuttan et al., 2015; Randall et al., 2017; Yildirim et al., 2017), though findings are not necessarily consistent across studies (Fayad et al., 2017). Dental anxiety is a complex issue, and the relationship between one factor may be influenced by other factors. Possible reasons for discrepancies in findings include differences in sampling methods, the recruitment of clinical samples, and the use of different dental anxiety measurements. Further research is needed to better understand the relationship between dental anxiety and various factors.

Adverse childhood experiences (ACEs) refer to traumatic events that occur before the age of 18. They include a broad range of stressful or disruptive events such as (i) childhood maltreatment (which can take various forms of neglect and abuse, including mental, physical or sexual), (ii) family dysfunction (such as living with household members with alcohol or drug addiction, mental illness, suicidal tendencies, a history of incarceration, experience of parental death, separation or divorce, and witnessing domestic violence), and (iii) violence outside the home (including being bullied, witnessing community violence, and exposure to collective violence) (World Health Organization, 2020). It is estimated that ACEs have a high global prevalence ranging from 41 to 97% (Carlson et al., 2020), which is a significant concern due to their long-term detrimental impact on individuals’ health and well-being. Research has shown significant negative effects of ACEs on both physical and mental health, as well as behavioral problems and social issues (Bethell et al., 2014; Kalmakis and Chandler, 2015; Hughes et al., 2017). Critically, ACEs have also been found to play a significant role in the development of psychiatric conditions such as schizophrenia (Baldini et al., 2023) and bipolar disorder (Miola et al., 2023). Studies have revealed that early-life traumas can disrupt neurodevelopment, contributing to the pathogenesis of these severe mental illnesses. Furthermore, the relationship between ACEs and suicidal behaviors has been established, with individuals exposed to ACEs demonstrating a higher risk of suicidal ideation, self-harm, and suicide attempts (Dube et al., 2001). This highlights the importance of understanding the complex interplay between ACEs and severe psychiatric conditions, and the need for targeted interventions to address these issues.

ACEs have been found to negatively impact individuals’ physical health. Previous studies have shown that individuals with ACEs have a higher risk of developing chronic diseases such as heart disease, diabetes, cancer, and obesity (Monnat and Chandler, 2015; Wiss and Brewerton, 2020). Furthermore, research suggests that ACEs can weaken individuals’ immune systems, making them more susceptible to diseases (John-Henderson et al., 2020). Studies have also shown that individuals with a higher ACE score have a higher risk of sleep disturbances (Kajeepeta et al., 2015) and early death (Brown et al., 2009). Research has also shown that individuals with ACEs are at a higher risk of developing mental health issues later in life, such as depression, anxiety and other mood disorders, post-traumatic stress disorder (PTSD), or other trauma-related mental health issues (Kalmakis and Chandler, 2015; Hughes et al., 2017). Furthermore, ACEs have been associated with the development of undesirable behavioral and social issues, such as drug or substance addiction, smoking, criminal behavior, unprotected sexual activities, attempting suicide, having interpersonal difficulties, and struggling to build and maintain healthy relationships (Dube et al., 2001; Poole et al., 2018; Petruccelli et al., 2019).

Although many studies have investigated the risk factors of dental anxiety, most have been limited to considering only dental-related stimuli and experiences, overlooking factors beyond the dental field. Research suggests that non-dental traumatic experience may also contribute to the development of dental anxiety (De Jongh et al., 2006). To the best of our knowledge, no study has yet been conducted to investigate the impact of ACEs (i.e., experiences before the age of 18 years) on dental anxiety. The aim of this study was to explore the association between dental anxiety and ACEs in a sample of Chinese adults in Hong Kong. It was hypothesized that ACEs would be positively associated with dental anxiety. The findings of this study might contribute to the understanding of ACEs survivors and inform clinicians whether it is necessary to inquire about ACE history before initiating dental treatment.

## 2 Materials and methods

### 2.1 Study design

This was an observational cross-sectional survey study that investigated the relationship between dental anxiety and adverse childhood experience in adult. It was reported according to the STROBE statement, and was conducted in accordance with the Declaration of Helsinki. Ethical approval was obtained from the Institutional Review Board of the University of Hong Kong/Hospital Authority Hong Kong West Cluster (HKU/HA HKW IRB) (IRB reference number: UW 22–600) prior to the start of the study. Informed consent was embedded in the online survey.

### 2.2 Setting

The survey study was conducted in a web-based format. Subjects were recruited from a major university in Hong Kong. An online invitation with study flyers, weblink and quick response (QR) code were sent to university staff and students via mass e-mails with a request to participate on a voluntary basis. In order to maximize reach and response, individuals who received the invitation were encouraged to forward the invitation link to their friends who are currently affiliated with the university, as not everyone may receive or respond to mass e-mails. Respondents were provided with a link to an online questionnaire, which began by asking if they could read Chinese. If they indicated they could not, they were then directed to an English version. The recruiting period and data collection started in January 2023 and ended in June 2023. Participants needed to respond once without a follow-up.

### 2.3 Participants

Convenience and snowball sampling method was adopted. Subjects who were at least 18 years old were included. People were excluded if they were unable to read English or Chinese, or if they self-reported to have known psychological disorders such as Generalized Anxiety Disorder, depression, or any other psychiatric conditions listed in the DSM-V.

### 2.4 Outcome measures

The outcome variable in this study was dental anxiety. Dental anxiety was defined as the level of anxiety associated with dental-related setting that an individual experienced.

The independent variable was adverse childhood experiences (ACE). ACE was defined as some excessively demanding sources of stress or potential traumatic experiences that an individual suffered in their early life (World Health Organization, 2020). ACE in the current study was examined in two dimensions: (1) overall ACE exposure – total number of different ACEs that an individual was exposed to (i.e., cumulative ACEs); and (2) exposure to individual ACE components – independent ACEs that an individual was exposed to.

### 2.5 Measurement tools

#### 2.5.1 Adverse childhood experience

Exposure of childhood adversity during the first 18 years of life was examined by using the ACE-IQ (World Health Organization, 2020) and its validated Chinese version (Ho et al., 2019). This measure consists of 29 questions that fall into 13 categories of childhood adversities, namely (1) emotional neglect, (2) physical neglect, (3) emotional abuse, (4) physical abuse, (5) contact sexual abuse, (6) alcohol and/or drug abuser in the household, (7) Someone chronically depressed, mentally ill, institutionalized or suicidal, (8) incarcerated household member, (9) one or no parents, parental separation or divorce, (10) household member treated violently, (11) bulling, (12) witnessing community violence, and (13) exposure to war or collective violence. Binary version was adopted as the scoring method in this study. Any experience to each childhood adversity was identified as an “exposure” regardless of the frequency of the exposures (Supplementary Figure S1). Each question was offered with one of the three response options: (1) “Yes” or “No”; (2) a 4-point Likert scale ranging from “Never” to “Many times”; and (3) a 5-point Likert scale ranging from “Never” to “Always.” Finally, the responses to all questions were dichotomized into “exposed” or “not exposed.” The total number of exposures were summated to create a cumulative ACE score ranging from 0 (No ACE exposure) to 13 (exposure to all ACE categories). The ACE-IQ demonstrated a good internal consistency, with Cronbach’s alpha of 0.83 and good content validity (Ho et al., 2019). A brief description of each ACE exposure, as described by World Health Organization (2020), is listed in Supplementary Table S1.

#### 2.5.2 Dental anxiety

Dental anxiety was assessed by the MDAS (Humphris et al., 1995) and its validated Chinese version (Lin et al., 2021). The MDAS is a self-reported measure that consists of five hypothetical questions concerning feelings of specific dental-related situations: (1) treatment tomorrow; (2) waiting room; (3) tooth drilled; (4) teeth scaled and polished; and (5) local anesthetic injection. The response to each question is in the format of a 5-point Likert scale that ranges from “not anxious” to “extremely anxious.” The total score of MDAS ranges from 5 to 25, with a higher score indicating a higher level of dental anxiety. A cut-off value of 19 and above was empirically determined as having a high level of dental anxiety (Humphris et al., 1995, 2009). MDAS has demonstrated high levels of internal consistency (Humphris et al., 1995, 2009; Lin et al., 2021).

Dental anxiety was also examined by the DFS (Kleinknecht et al., 1984) and its validated Chinese version (Liang et al., 2006). The DFS is a 20-item self-administered questionnaire that assesses (1) avoidance behavior; (2) physiological response; (3) specific dental-related stimulation or situation; and (4) the overall level of fear. Response format follows a 5-point Likert scale ranging either from “never” to “nearly every time” or “not at all” to “very much.” The total score of DFS ranges from 20 to 100, with a higher score indicating a higher level of dental fear. Without an official cut-off value, a cut-off value of 59 or above was suggested as having a high level of dental fear (Skaret et al., 1999). The DFS had good reliability and validity (Kleinknecht et al., 1973, 1984; Skaret et al., 1999; Liang et al., 2006).

### 2.6 Data collection

All data was obtained via an online survey system. Participants were instructed to self-complete an anonymous structured online questionnaire.

### 2.7 Sample size calculation

A previous study on adults has reported a correlation coefficient of 0.2 between ACE score and health anxiety score (Reiser et al., 2014). We assumed that there would be a similar level of correlation between ACE score and dental anxiety. For a two-tailed test with an alpha of 0.05 and power of 0.80, against the null hypothesis of zero correlation, the required sample size was calculated to be 150. With a response rate of 90%, at least 170 adults would be recruited.

### 2.8 Statistical analysis

Both the relationship of each individual ACE components (13 categories) and the total ACE score (cumulative number of ACEs that ranged from 0 to 13) with dental anxiety were assessed. The collected data was analyzed with the Statistical Package for Social Sciences (IBM Corp. Released 2019. IBM SPSS Statistics for Windows, Version 26.0. Armonk, NY: IBM Corp). Respondents with any missing data would be excluded from analysis (eventually there was no missing data). Descriptive statistics were computed for demographic information. Continuous variables were summarized as a mean or median value with their standard deviations, while the frequency counts of categorical variables were reported. To explore the relationships between cumulative ACEs, individual ACE components and dental anxiety (MDAS and DFS score), Pearson correlations, linear regression and logistic regression were conducted. The regression analyses did not include any factors as confounding variables. ANOVA was conducted to reveal if there were any differences in the mean age, MDAS and DFS scores between groups with 0 ACE, 1–3 ACEs, and ≥ 4 ACEs. Similarly, chi-squared tests were conducted to compare demographic data between these groups.

## 3 Results

### 3.1 Descriptive statistics

All participants (*n* = 171) were included in the analysis with no missing data. Out of the 171 participants, 123 (71.9%) were females, and 48 (28.1%) were males. The mean (SD) age was 27.6 (10.2) years, with a full range of 18–67. There were no significant differences in demographic data between participants with and without ACE exposure. Over half of the participants were students (52.1%, *n* = 89), whereas less than half of the participants were having a job (43.9%, *n* = 75). Most of the participants (91.8%, *n* = 157) were attending or have already completed higher education (i.e., from sub-degree to doctoral levels).

### 3.2 Dental anxiety and ACEs exposure of the study cohort

**Figure 1:**
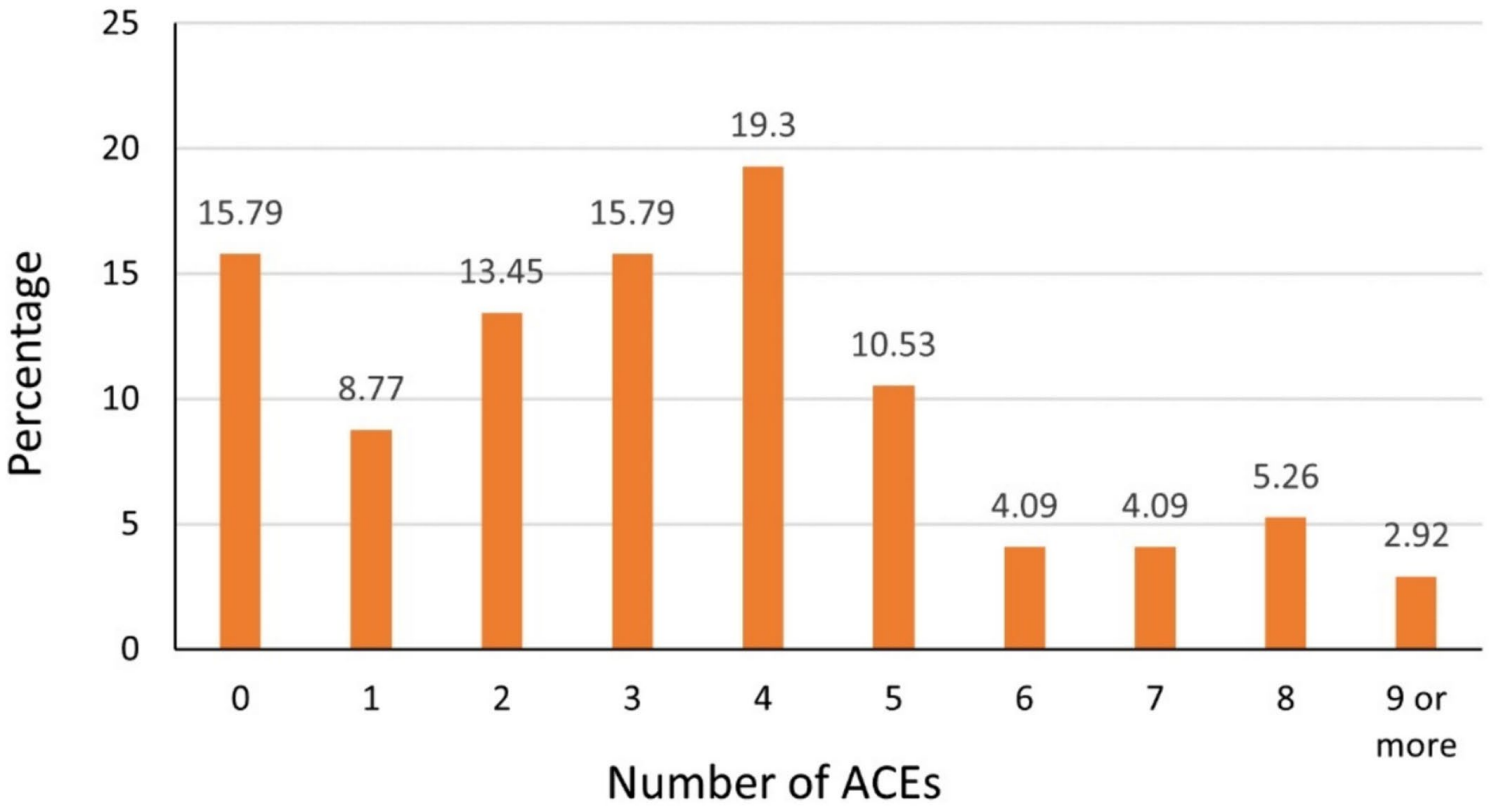
Percentage of 13 ACEs components in the study cohort.

Compared with the groups without ACE exposure or with 1–3 ACEs exposure, participants who reported four or more ACEs had significantly higher mean (SD) scores of MDAS (*p* = 0.010) and DFS (*p* = 0.005). Individuals who reported 4 or more ACEs were more likely to have higher dental anxiety scores when compared with individuals without ACEs exposure (Table 1). Overall, 84.2% (n = 144) of the participants were exposed to at least 1 of the 13 ACEs, and 46.2% were exposed to four or more (Figure 1). The prevalence of each individual ACE components ranged from 1.75% (Incarcerated household member) to 67.3% (Emotional abuse). On average, participants have been exposed to a mean (SD) of 3.37 (2.48) ACEs. Of the participants with ACEs, the ACE with highest prevalence was emotional abuse (67.5%), followed by domestic violence (62.5%), physical abuse (49.1%) and bullying (45%) (Table 2).

**Table 1:** Demographic data and other characteristics of the study cohort.

	Full sample (*n* = 171)		0 ACE (n = 27)		1–3 ACEs (*n* = 65)		≥4 ACEs (*n* = 79)		*p*-value^3^
Characteristics	*n*	%	*n*	%	*n*	%	*n*	%	
Age^1^									
Mean	27.58		30.26		26.57		27.49		0.290
(SD)	(10.24)		(16.69)		(7.83)		(10.14)		
Range	18–67		18–66		18–47		18–67		
Gender^2^									
Male	48	28.07	7	25.9	20	30.8	21	26.6	0.826
Female	123	71.93	20	74.1	48	69.2	58	73.4	
Marital status^2^									
Never married	132	77.19	18	66.7	50	76.9	64	81.0	0.308
Married	39	22.81	9	33.3	15	23.1	15	19.0	
Education attainment^2^									
Primary	1	0.58	0	0	0	0	1	1.3	0.485
Secondary	11	6.43	4	17.8	2	3.1	5	6.3	
Certificate/Diploma	2	1.17	0	0	2	3.1	0	0	
Associate/Sub-degree	9	5.26	1	3.7	2	3.1	6	7.6	
Bachelor degree	102	59.65	17	63.0	40	64.5	45	57.0	
Master’s degree	36	21.05	4	14.8	14	21.5	18	22.8	
Doctorate	10	5.85	1	3.7	5	7.7	4	5.1	
Economic status^2^									
Employed	75	43.86	10	37.0	31	47.7	34	43.0	0.578
Retired persons	4	2.34	1	3.7	1	4.5	2	2.5	
Students	89	52.05	16	59.3	33	50.8	40	50.6	
Others	3	1.75	0	0	0	0	3	3.8	
Mean score of dental anxiety measures^1^									
MDAS	11.94	(4.35)	11.67	(3.82)	10.08	(3.73)	12.97	(4.78)	0.010^4^
DFS	43.73	(16.85)	42.70	(16.90)	38.94	(13.55)	43.73	(16.85)	0.005^4^

Items with zero subjects were not presented in the table, including (i) Marital status: widowed, divorced /separated; (ii) Education attainment: no schooling, pre-primary; (iii) Economic status: unemployed, home-makers. MDAS, Modified Dental Anxiety Scale; DFS, Dental Fear Survey.^1^Continuous data are reported as the mean (SD).^2^Categorical data are reported as the number (n) and percentage (%) of participants.^3^*p*-value were computed to compare the differences between three groups (i.e., Group 1 = 0 ACE; Group 2 = 1–3 ACEs; and Group 3 = ≥4 ACEs).^4^In *post hoc* Tukey test, significant group differences were found for the following pairs: (i) MDAS: Group 3 > Group 2; (ii) DFS: Group 3 > Group 2.

**Table 2:** Prevalence of adverse childhood experiences (ACEs).

	Full sample (*n* = 171)	
	*n*	%
Childhood maltreatment		
Emotional neglect	24	14.04
Physical neglect	28	16.37
Emotional abuse	115	67.25
Physical abuse	84	49.12
Contact sexual abuse	21	12.28
Family dysfunction		
Alcohol and/or drug abuser in the household	8	4.68
Someone chronically depressed, mentally ill, institutionalized or suicidal	16	9.36
Incarcerated household member	3	1.75
One or no parents, parental separation or divorce	27	15.79
Household member treated violently	107	62.57
Violence outside the home		
Exposure to peer violence (bullying)	77	45.03
Witnessing community violence	54	31.58
Exposure to war or collective violence	12	7.02

### 3.3 Relationship between adverse childhood experiences and dental anxiety

Pearson correlation test was conducted to assess the linear relationship between the number of ACEs (as measured by the ACE-IQ questionnaire), and dental anxiety (as measured by MDAS and DFS). The number of ACEs was positively correlated with MDAS scores (*r* = 0.169, *p* = 0.027) and DFS scores (*r* = 0.253, *p* < 0.001) (Supplementary Table S2).

Linear regression has revealed that the number of ACEs significantly correlated to dental anxiety score (i.e., MDAS and DFS). For MDAS, test result was significant: *F*(1,169) = 4.962, *p* = 0.027, *R*^2^ = 0.029. A participant’s expected MDAS score was equal to 10.942 + 0.297*(number of ACEs). In other words, a participant’s MDAS score would increase by 0.297 for every additional exposure to ACEs. Test result was also significant for DFS: *F*(1,169) = 11.561, *p* < 0.001, *R*^2^ = 0.064. A participant’s expected DFS score was equal to 37.928 + 1.721*(number of ACEs). In other words, if a participant had one more exposure to ACE, then his/her DFS score would be increased by 1.721.

Logistic regression was performed to ascertain the effects of number of ACEs on the likelihood that participants had high dental anxiety as measured by MDAS and DFS. The logistic regression model was statistically significant when MDAS was measured: χ^2^(1) = 10.575, *p* < 0.005. The model explained 14.4% (Nagelkerke *R*^2^) of the variance in high dental anxiety and correctly classified 92.4% of cases. The odds of an individual having high dental anxiety would be increased by 43% (OR = 1.43, 95% CI = 1.146–1.786) for every additional exposure to ACEs (*p* = 0.002). When dental anxiety was measured by DFS, the logistic regression model was also statistically significant: χ^2^(1) = 9.146, *p* < 0.005. The model explained 8.2% (Nagelkerke *R*^2^) of the variance in high dental anxiety and correctly classified 80.7% of cases. The odds of an individual having high dental anxiety would be increased by 26% (OR = 1.26, 95% CI = 1.08–1.46) for every additional exposure to ACEs (*p* = 0.003).

Logistic regression was performed to assess the effects of individual ACEs (i.e., 13 categories) on the likelihood of having high dental anxiety (Table 3). Participants who were exposed to physical neglect before 18 years of age were 5.30 times (MDAS: OR = 5.30, 95% CI = 1.63–17.24, *p* = 0.006) or 3.91 times (DFS: OR = 3.91, 95% CI – 1.64 – 9.35, *p* = 0.002) more likely to have high dental anxiety. Participants who had childhood sexual abuse were 3.69 times (MDAS: OR = 3.68, 95% CI = 1.02–13.27, *p* = 0.046) or 2.80 times (DFS: OR = 2.80, 95% CI = 1.06–7.43, *p* = 0.038) more likely to have high dental anxiety. Exposure to collective violence and living with alcohol and/or drug abusers were also associated with high dental anxiety as shown by both MDAS and DFS models. Meanwhile, the MDAS model additionally identified emotional neglect as an associated factor, whereas being bullied and exposure to community violence were identified by the DFS model (Table 3).

**Table 3:** Prevalence and odds ratios for the relationship between individual ACE components and dental anxiety.

			MDAS			DFS		
	*n*	%	OR	95% C.I.	*p*-value	OR	95% C.I.	*p*-value
Emotional neglect								
Yes	24	14	4.572	(1.356, 15.423)	0.014	1.750	(0.662, 4.624)	0.259
No*	147	86						
Physical neglect								
Yes	28	16.4	5.299	(1.629, 17.240)	0.006	3.913	(1.637, 9.353)	0.002
No*	143	83.6						
Emotional abuse								
Yes	115	67.3	1.683	(0.444, 6.373)	0.444	1.278	(0.566, 2.886)	0.555
No*	56	32.7						
Physical abuse								
Yes	84	49.1	2.490	(0.736, 8.421)	0.142	1.738	(0.816, 3.700)	0.152
No*	87	50.9						
Contact sexual abuse								
Yes	21	12.3	3.686	(1.024, 13.269)	0.046	2.803	(1.058, 7.427)	0.038
No*	150	87.7						
Alcohol and/or drug abuser in the household								
Yes	8	4.7	9.180	(1.914, 44.029)	0.006	7.389	(1.673, 32.627)	0.008
No*	163	95.3						
Someone chronically depressed, mentally ill, institutionalized or suicidal								
Yes	16	9.4	3.346	(0.817, 13.699)	0.093	1.894	(0.612, 5.861)	0.268
No*	155	90.4						
Incarcerated household member								
Yes	3	1.8	6.500	(0.549, 76.940)	0.138	8.182	(0.008, 0.815)	0.090
No*	168	98.2						
One or no parents, parental separation or divorce								
Yes	27	15.8	2.609	(0.741, 9.179)	0.135	1.450	(0.558, 3.766)	0.446
No*	144	84.2						
Household member treated violently								
Yes	107	62.6	1.378	(0.406, 4.670)	0.607	1.015	(0.471, 2.190)	0.969
No*	64	37.4						
Exposure to peer violence (Bullying)								
Yes	77	45.0	2.978	(0.880, 10.078)	0.079	2.143	(1.005, 4.571)	0.049
No*	94	55.0						
Witnessing community violence								
Yes	54	31.6	2.755	(0.879, 8.637)	0.082	2.527	(1.178, 5.419)	0.017
No*	117	68.4						
Exposure to war/collective violence								
Yes	12	7.0	4.967	(1.159, 21.281)	0.031	4.483	(1.349, 14.899)	0.014
No*	159	93.0						

*Reference category.

## 4 Discussion

The objective of the present study was to investigate the relationship between adverse childhood experiences (ACEs), including both cumulative ACEs and individual types of ACE components, and dental anxiety in adulthood. Building upon prior research (Reiser et al., 2014), we hypothesized that there would be significant positive associations between ACEs and dental anxiety. The findings of this study support our hypothesis, indicating that exposure to ACEs is indeed associated with increased dental anxiety. Specifically, when ACEs were assessed cumulatively, participants who reported a higher number of ACEs exhibited elevated dental anxiety scores, as measured by MDAS and DFS. Furthermore, our results revealed a significant association between the number of ACEs and the risk of developing high dental anxiety. In addition to the cumulative number of ACEs, our findings also suggest that specific types of ACE components, such as sexual abuse, physical neglect, collective violence, and living with alcohol and/or drug abuse, were independently associated with high level of dental anxiety.

Although the association between ACEs and negative health outcomes has been extensively explored (Hughes et al., 2017; Petruccelli et al., 2019), no previous study has specifically investigated the impact of ACEs on dental anxiety. Therefore, the present study represents a novel contribution by providing a comprehensive examination of both the cumulative and independent roles of ACEs in relation to dental anxiety. Our findings revealed that a significant proportion (84%) of the participants in this study reported exposure to at least one ACE. This prevalence is consistent with the results of a previous study that examined the patterns of ACE exposure in young adults in Hong Kong using the ACE-IQ questionnaire (74%) (Ho et al., 2019). Furthermore, our findings align with international norms, as a recent systematic review demonstrated that 75% of participants across 61 studies had experienced ACEs (Pace et al., 2022).

The results of the present study provide evidence that dental anxiety is significantly associated with the number of ACEs, which is consistent with findings from previous studies examining mental health outcomes. Several similar studies that employed the ACE-IQ questionnaire to investigate the impact of childhood adversity on health outcomes have reported significant associations between ACE exposure (both cumulative and individual ACEs) and increased odds of anxiety disorders (Subramaniam et al., 2020), psychological distress (Agbaje et al., 2021), and health-related quality of life (Cohrdes and Mauz, 2020). Besides, findings from a study that utilized a different form of ACE measurement also suggested a significant association between ACEs and health anxiety (Reiser et al., 2014).

Furthermore, specific components of ACEs, such as physical neglect and emotional neglect, were identified in this study as factors that increase the likelihood of high dental anxiety. The association between neglect as a form of childhood maltreatment and high dental anxiety can be explained by the development of early maladaptive schemas. Early maladaptive schemas refer to emotional and cognitive patterns that form early in life and continue to be elaborated and repeated throughout life, often to a dysfunctional extent (Young, 1990). A comprehensive review of early maladaptive schemas can be found in (Young et al., 2006). Individuals who have experienced neglect as a form of childhood maltreatment may develop characterological problems and personality disorders (Young, 1999). Consequently, they may exhibit negative cognitive styles or perceptual disturbances, such as being overly vigilant and suspicious of others and having a sense of shame (Rose and Abramson, 1992; Ered and Ellman, 2019; Loewy et al., 2019). These factors can in turn influence their behaviors, leading to social withdrawal and distancing from others (Alink et al., 2012; Maier et al., 2020). These behaviors have been well-established as factors associated with dental anxiety (Moore et al., 1993; De Jongh et al., 2006).

In addition, this study found that sexual abuse was associated with an increased likelihood of high dental anxiety. This can be explained by the perception individuals may have of dentists and dental situations following exposure to ACEs. Sensory stimuli associated with dental treatments, such as sight of hands, sounds of breathing from the dentist, taste and smell of gloves, and sensations of dental instruments and fingers inside the mouth, might trigger memories associated with their experiences of sexual abuse or even serve as a repetition of the abuse itself (Leeners et al., 2007; Fredriksen et al., 2020). Besides, victims of sexual abuse often exhibit poor self-image, fear of intimacy, and difficulties in forming trusting relationships (Davis and Petretic-Jackson, 2000), which can contribute to feelings of powerlessness and a lack of control (Chouliara et al., 2014). These factors have been shown to be associated with dental anxiety (Willumsen, 2004; Scandurra et al., 2021).

The measurement of ACEs and how they are measured is crucial in addressing research questions. A recent meta-analysis by Hughes et al. (2017), which examined 37 studies investigating the relationship between ACEs and health outcomes, found a diverse variation in the items used to classify ACEs across the studies. Some studies measured childhood adversity through aspects of “family dysfunction” and “childhood maltreatment experiences,” while others also included the aspect of “violence exposure.” The inconsistency in measuring childhood adversity may lead to inconsistent and inconclusive results, highlighting the importance of standardizing the measurement approach in ACE research.

The measurement of ACEs often involves a cumulative risk approach, which uses a sum score to assess an individual’s exposure to adverse experiences (Hughes et al., 2017; Petruccelli et al., 2019). This approach assumes that the accumulation of risk factors negatively impacts development and increases the likelihood of negative health outcomes. The more risk factors an individual is exposed to, the higher the prevalence of clinical problems or the worse the outcome (Sameroff, 2000; Appleyard et al., 2005; Evans et al., 2013). While this cumulative approach provides a general picture of the impact of ACEs and their potential to predict negative outcomes, it may not be adequate or clinically meaningful in practice when individualized intervention is desired. This is because the distinctive nature and effect of each individual’s childhood adversity may be overlooked. Hence, while the use of a cumulative ACE score can identify high-risk individuals and facilitate early intervention strategies, it may not provide tailored intervention for every individual. It is important to consider alternative approaches that capture the unique experiences and effects of individual ACEs. For example, our findings found that dental anxiety was associated with only sexual abuse, but not emotional abuse, and physical abuse.

The present study has several potential limitations that should be acknowledged. Firstly, there is the possibility of response bias, as participants may have exaggerated or underreported their self-reported measures of ACEs and dental anxiety. This could occur when respondents overstated their negative feelings and experiences, influencing their responses. Conversely, respondents might have difficulty recalling past events or might hesitate to disclose their experiences and behaviors, leading to misclassification or misidentification of exposure to the outcome measures. This limitation could potentially result in an over- or underestimation of the strength of the relationships between ACEs and dental anxiety. Secondly, the sample in the present study was limited to university students and staff. As a result, caution should be exercised when generalizing the findings to the general population, as the external validity of the results might be limited.

From another perspective, the prevention of ACEs is a critical area of focus for researchers, policymakers, and practitioners. There are several potential future directions that could be explored in order to advance the field and contribute to the development of more effective prevention strategies. One important aspect of ACE prevention is early intervention and support for families and children at risk. This can be achieved through the provision of resources and education for new parents, offering parenting classes and support groups, and implementing home visitation programs for families in high-risk situations. A comprehensive approach to prevention must also include expanding access to mental health services for children and families. This may involve increasing funding for mental health care, training more mental health professionals, and integrating mental health services into schools and community centers. By preventing the development of ACEs, it is hoped that young people in future generations will generally have a lower level of dental anxiety, so that they can arrange prompt dental visits to maintain their oral health and oral health-related quality of life.

In conclusion, our study found a positive association between ACEs and dental anxiety, with certain types of individual ACEs showing increased odds of high dental anxiety. The results from MDAS and DFS were not consistent, indicating that contextual differences may exist between these measurement tools of dental anxiety. Understanding the relationship between ACEs and dental anxiety is important not only for patients and dental practitioners in terms of screening target patients, developing treatment plans, and selecting appropriate interventions tailored to individual needs, but also for society in providing services adapted to the local context. Dental practitioners should inquire about and understand a patient’s ACE history as part of their background information and show empathy towards ACE survivors with interactions deemed comfortable by them.

## Data availability statement

The original contributions presented in the study are included in the article/Supplementary material, further inquiries can be directed to the corresponding author.

## Ethics statement

The studies involving humans were approved by Institutional Review Board of the University of Hong Kong/Hospital Authority Hong Kong West Cluster. The studies were conducted in accordance with the local legislation and institutional requirements. The participants provided their written informed consent to participate in this study.

## Author contributions

NW: Conceptualization, Data curation, Formal analysis, Investigation, Writing – original draft, Writing – review & editing. AY: Data curation, Writing – original draft, Writing – review & editing. CM: Supervision, Writing – original draft, Writing – review & editing. YL: Supervision, Writing – original draft, Writing – review & editing.
