# Supplementary material for: Adverse childhood experiences and dental anxiety among Chinese adults in Hong Kong: a cross-sectional study
Source: Front Psychol. 2024 May 22;15:1372177. doi: 10.3389/fpsyg.2024.1372177 (PMC11150826; doi:10.3389/fpsyg.2024.1372177)
Supplement: Supplementary file 1 [file Table_1.DOCX]

**Supplementary Table 1. Brief Description of ACE Exposure.**

| **ACE** | | **Description** |
| --- | --- | --- |
| Childhood Maltreatment | | |
|  | 1. Emotional neglect | Parents or guardians did not understand the problems and worries of their children; parents or guardians did not really know what their children were doing when they were not at school or work |
|  | 2. Physical neglect | Parents or guardians did not give enough food even they could easily have done so; too drunk or intoxicated by drugs in nurturing; did not send their children to school when it was available. |
|  | 3. Emotional abuse | Being yelled at, screamed at, sworn at, insulted or humiliated by parents, guardian or other household members; being threatened to or actually abandoned or being thrown out of the house. |
|  | 4. Physical abuse | Being spanked, slapped, kicked, punched or beaten up by a parent, guardian or other household member; being hit or cut with an object such as a stick, bottle, club, knife, whip by a parent, guardian or other household member. |
|  | 5. Contact sexual abuse | Being touched or forced to touch others’ body in a sexual way reluctantly; being attempted or actually had oral, anal or vaginal intercourse with others reluctantly. |
| Family Dysfunction | | |
|  | 6. Alcohol and / or drug abuser in the household | Lived with a household member who was a problem drinker, alcoholic, misused street or prescription drugs user. |
|  | 7. Someone chronically depressed, mentally ill, institutionalized or suicidal | Lived with a household member who was depressed, mentally ill or suicidal. |
|  | 8. Incarcerated household member | Lived with a household member who was ever sent to jail or prison. |
|  | 9. One or no parents, parental separation or divorce | Parents were separated or divorced; mother, father or guardian died. |
|  | 10. Household member treated violently | Saw or heard a parent or household member in home being yelled at, screamed at, sworn at, insulted, humiliated, slapped, kicked, punched, beaten up, or hit or cut by an object such as a stick, bottle, club, knife, whip etc. |
| Violence Outside the Home | | |
|  | 11. Exposure to peer violence (Bullying) | Being bullied (i.e. A young person or group of young people say or do bad and unpleasant things to another young person; a young person is teased a lot in an unpleasant way; a young person is left out of things on purpose). |
|  | 12. Witnessing community violence | Seen or heard your neighborhood or community (not in your home or on TV, movies, or the radio) being beaten up, stabbed, shot, or threatened with a knife or gun in real life. |
|  | 13. Exposure to war or collective violence | Being forced to go and live in another place; experienced the deliberate destruction of home; family member or individual being killed, beaten up by soldiers, police, militia or gangs due to the following experiences: events involved collective violence, including wars, terrorism, political or ethnic conflicts, genocide, repression, disappearances, torture and organized violent crime such as banditry and gang warfare. |

**Supplementary Table 2. Correlation of ACE and Dental Anxiety.**

| Measure | 1 | 2 | 3 |
| --- | --- | --- | --- |
| 1. ACE-IQ (Total no. of ACEs) | - |  |  |
| 2. MDAS | .169* | - |  |
| 3. DFS | .253** | .874** | - |

* = Correlation is significant at the 0.05 level (2-tailed).

** = Correlation is significant at the 0.01 level (2-tailed).

ACE-IQ = Adverse Childhood Experiences International Questionnaire; MDAS = Modified Dental Anxiety Scale; DFS = Dental Fear Survey.


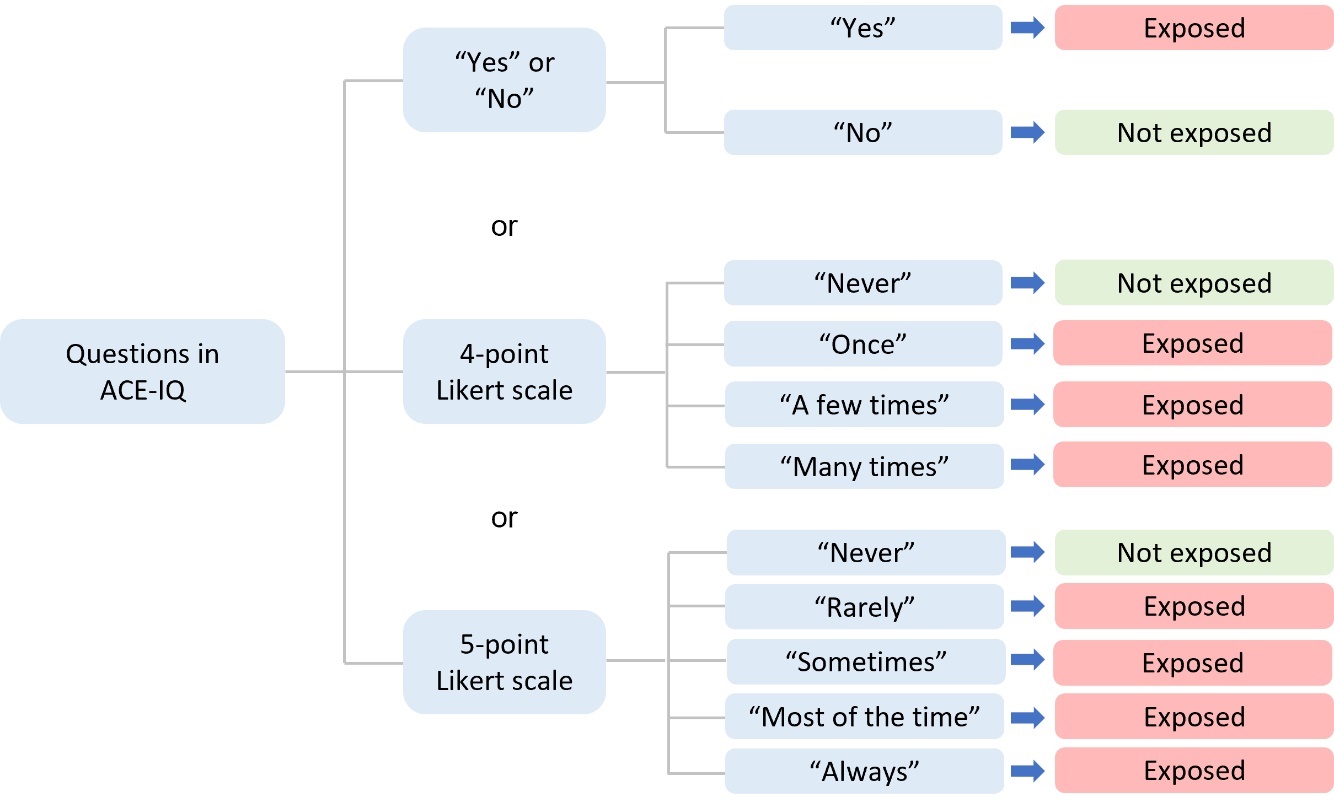


**Supplementary Figure 1.** Scoring method of Adverse Childhood Experiences International Questionnaire (ACE-IQ)
